# Supplementary material for: The experience of people living with heart failure in Ethiopia: A qualitative descriptive study
Source: PLoS One. 2024 Oct 24;19(10):e0310600. doi: 10.1371/journal.pone.0310600 (PMC11500853; doi:10.1371/journal.pone.0310600)
Supplement: S1 File — (PDF) [file pone.0310600.s001.pdf]

<Files\\Data\\P01> - § 5 references coded [6.12% Coverage]

Reference 1 - 1.59% Coverage

I was shocked when I heard my diagnosis; I didn’t expect that i can survive; i was saying bye to my families.

Reference 2 - 1.31% Coverage

So, the feeling of tiredness makes me to feel worse, and i feel better when I'm resting.

Reference 3 - 0.72% Coverage

My life is not as it was before, it has changed.

Reference 4 - 1.50% Coverage

Your heart could stop working and you could die if you try to perform any activity beyond your ability.

Reference 5 - 0.99% Coverage

since I started taking medications, I have noticed some improvement.

<Files\\Data\\P02> - § 3 references coded [6.43% Coverage]

Reference 1 - 1.61% Coverage

I used to eat whatever I wanted, but now I have advised to avoid fatty foods and meat.

Reference 2 - 3.18% Coverage

Now everything is better than before. I have seen many patients with worse symptoms as compared to mine. So that am thankful, I'm feeling good and better than many people.

Reference 3 - 1.65% Coverage

The worst is the feeling of chest pain, depression, and a generalized feeling of fatigue.

<Files\\Data\\P03> - § 6 references coded [10.79% Coverage]

Reference 1 - 2.07% Coverage

I don't feel bad and said, "this condition happened to me with the will of God". So, I accepted. Then, I received my medical care, and I was hospitalized for a couple of days.

Reference 2 - 0.91% Coverage

I know that God is with me, so I didn't feel bad when I heard my diagnosis.

Reference 3 - 0.54% Coverage

Now I'm feeling good after I start medications

Reference 4 - 1.51% Coverage

I feel discouraged sometimes drugs are searched for and disappeared; if I don't take my medicine, there will be some congestion.

Reference 5 - 2.83% Coverage

Now I follow my treatment and specially the last 6 months i think I've recovered so much. I was hospitalized at ICU…there were times when me and my families felt like am hopeless and dying, but amazingly I recovered and feeling better now.

Reference 6 - 2.93% Coverage

Visiting the hospital every month and taking medicine is also the worst. You aren't allowed to eat everything you want, there are limitations on every part of your life, and if you're relaxing a little on your treatment, you're gone. This is boring

<Files\\Data\\P04> - § 4 references coded [7.41% Coverage]

Reference 1 - 2.15% Coverage

When I heard my diagnosis, I didn't think I would survive because it's a serious and terrifying illness, but I'm grateful that I'm still alive today

Reference 2 - 2.17% Coverage

I would become angry and agitated if my family and I had a minor argument. This made my problems worse. I feel better if there's nothing to upset me.

Reference 3 - 1.44% Coverage

I used to think that I am going to die, but now I feel much better after I started my medications.

Reference 4 - 1.64% Coverage

I am unable to eat what I want. I used to eat everything before this illness, but now I only eat certain things.

<Files\\Data\\P05> - § 3 references coded [6.27% Coverage]

Reference 1 - 1.48% Coverage

I was shocked to hear the diagnosis and thought I wouldn't be able to survive.

Reference 2 - 3.39% Coverage

There were times when I thought I was going to die, and you know when that happens, you are totally lost. However, I am now feeling better because of the medical care I received.

Reference 3 - 1.40% Coverage

I feel better after I start taking my medications, yes! I feel better.

<Files\\Data\\P06> - § 6 references coded [9.20% Coverage]

Reference 1 - 1.64% Coverage

I was horrified when I first heard of my diagnosis and could not sleep at night because I was working there and worried about losing my job.

Reference 2 - 0.95% Coverage

I have seen improvement after I started my follow up and taking medication here.

Reference 3 - 0.86% Coverage

I am completely different person in all aspects. I am not the same person.

Reference 4 - 2.65% Coverage

I'm grateful because a lot of others have much worse problems than I do. Many people are unable to visit this facility for their regular follow-up appointment. When you see something worse, you are grateful for your condition.

Reference 5 - 1.15% Coverage

The worst is the shortness of breath; it's particularly challenging if you have breathing problems

Reference 6 - 1.95% Coverage

The worst aspect of this diseases is it makes you to be dependent on others. You feel awful when you realize you used to help others and are now dependent on others.

<Files\\Data\\P07> - § 8 references coded [9.55% Coverage]

Reference 1 - 2.47% Coverage

I was not surprised when I received my diagnosis since I know that a person with diabetes and high blood pressure will eventually be vulnerable to heart disease. I calmed and started to follow the physicians' recommendations.

Reference 2 - 0.65% Coverage

The worst is when a medication disappears off the market.

Reference 3 - 1.15% Coverage

My condition has been better since my angiography. I can now go on walks and perform small tasks at home.

Reference 4 - 1.21% Coverage

I never got out of bed before, but now that I can visit the hospital and seek medical treatment, I feel better.

Reference 5 - 1.75% Coverage

If your heart stops, it's like your car's engine; you can't move. This condition is dangerous… if you don't take care of your self … your life will be miserable.

Reference 6 - 0.32% Coverage

I have noticed good progress.

Reference 7 - 1.29% Coverage

I thought I wouldn't be improved at all. But I have seen changes in my health status after starting my follow up care.

Reference 8 - 0.72% Coverage

The worst is when there is no one nearby that can provide support.

<Files\\Data\\P08> - § 5 references coded [10.32% Coverage]

Reference 1 - 1.45% Coverage

I've never had such a terrible illness, so when I heard of my diagnosis, I was shocked. I thought I am dying….so a bit scary.

Reference 2 - 1.17% Coverage

The thought of what would happen to my family if I passed away from this illness deeply disturbed me.

Reference 3 - 4.96% Coverage

I used to work hard and pay my bills, but now I can't even lift a heavy object. My medical condition prevents me from working, so my wife is now responsible for paying my bills because I am unable to do so. While I tried to work, I fell. So, my quality of life decreased… you know before to my illness, I was able to take care of my family's needs, was socially active, and worked hard. However, after becoming ill, all has changed.

Reference 4 - 0.46% Coverage

After this illness my life has changed.

Reference 5 - 2.28% Coverage

I don’t have nothing to say Good. I haven't experienced any significant improvement in my life since being diagnosed with heart failure; I'm out of my life, and everything right now is gloomy for me.

<Files\\Data\\P09> - § 3 references coded [7.41% Coverage]

Reference 1 - 3.69% Coverage

I was shocked when I heard my diagnosis because I had never been ill before but after some time, I calmed and accepted my diagnosis.

Reference 2 - 2.44% Coverage

When I am thankful to God, I feel better. I pay more attention to what the doctor says:

Reference 3 - 1.28% Coverage

being unwell and tired are the worst feelings.

<Files\\Data\\P10> - § 3 references coded [3.16% Coverage]

Reference 1 - 1.56% Coverage

I wasn't shocked to hear of my diagnosis and declared that it had come happened as per God's will.

Reference 2 - 0.38% Coverage

Depression is the worst.

Reference 3 - 1.23% Coverage

The worst is that I'm unable to eat what I want and can't walk around easily.

<Files\\Data\\P11> - § 6 references coded [13.44% Coverage]

Reference 1 - 2.90% Coverage

The worst feeling is realising that I must always take medications for this illness and that I am not performing at the same level as my friends. I am tired of taking my pills daily.

Reference 2 - 3.83% Coverage

My lifestyle is not as it was before. You should deal with it. I was doing all sorts of activities without difficulty… you know what ...i did everything at home, but now I have limitations, a heavy activity makes me to feel tired. It is boring

Reference 3 - 0.88% Coverage

I am frustrated having to take medications all the time.

Reference 4 - 1.31% Coverage

In comparison to the last time, I feel good, and I think I have made good progress.

Reference 5 - 2.00% Coverage

The worst aspect of heart failure is that you could experience abrupt onset of symptoms, such as a headache or another symptom.

Reference 6 - 2.52% Coverage

The cost of medical care, needing to take medication and have regular check-ups, as well as feeling exhausted, are some of the worst effects of heart failure.

<Files\\Data\\P12> - § 3 references coded [2.87% Coverage]

Reference 1 - 0.63% Coverage

After I knew my condition, I was anxious.

Reference 2 - 1.21% Coverage

I was unable to sleep during the night due to my anxiety. I thought I would die.

Reference 3 - 1.03% Coverage

since I started taking the medication, thanks to God, I feel better.

<Files\\Data\\P13> - § 4 references coded [2.54% Coverage]

Reference 1 - 0.25% Coverage

I felt hopeless

Reference 2 - 1.25% Coverage

The worst is am dependent on my family and the symptoms specially my palpitation.

Reference 3 - 0.54% Coverage

There is limitation in all aspects.

Reference 4 - 0.51% Coverage

The good thing is I am improving.

<Files\\Data\\P14> - § 3 references coded [6.67% Coverage]

Reference 1 - 2.57% Coverage

I was shocked when I heard of my diagnosis since I have two kids and feel like how they would survive if I'm leaving them; I was worried about them, not about myself.

Reference 2 - 2.68% Coverage

Since I started taking the medication, I am feeling better. There is a good improvement as compared to before. I believe it is helping me in getting better from my symptoms.

Reference 3 - 1.42% Coverage

I am better than the previous times. I have seen many people in the OPD with worse symptoms.

<Files\\Data\\P01> - § 2 references coded [1.37% Coverage]

Reference 1 - 0.35% Coverage

I feel tired and fatigue

Reference 2 - 1.02% Coverage

I don't like to hear heavy noise, I'm worried. I usually being alone.

<Files\\Data\\P02> - § 3 references coded [4.57% Coverage]

Reference 1 - 1.31% Coverage

My legs are oedematous, and I feel dizziness and fatigue and exhausted.

Reference 2 - 0.92% Coverage

occasionally, I feel bad because of my depression.

Reference 3 - 2.33% Coverage

I can't even perform simple tasks like washing my socks and gardening. This makes me to feel hopeless, ultimately depressed.

<Files\\Data\\P03> - § 1 reference coded [0.11% Coverage]

Reference 1 - 0.11% Coverage

fatigued.

<Files\\Data\\P04> - § 4 references coded [4.48% Coverage]

Reference 1 - 2.31% Coverage

I have chest pain; fatigue; dyspnoea, abdominal pain; leg swelling; and decreased appetite. I have difficulty of breathing and I can't walk for long distance.

Reference 2 - 0.77% Coverage

My symptoms worsen when I walk outside in hot weather

Reference 3 - 0.83% Coverage

my disease makes me feel so depressed and affect my life.

Reference 4 - 0.57% Coverage

I also feel overwhelmed and helpless.

<Files\\Data\\P05> - § 4 references coded [3.41% Coverage]

Reference 1 - 1.72% Coverage

I still experience palpitations, shortness of breathing, and fatigue when I do heavy job.

Reference 2 - 0.36% Coverage

anxious and worried

Reference 3 - 1.04% Coverage

I feel down because I can't work as hard as I used to.

Reference 4 - 0.28% Coverage

I stay at home

<Files\\Data\\P06> - § 5 references coded [4.60% Coverage]

Reference 1 - 0.77% Coverage

I have fatigue, shortness of breathing, and sometimes dry cough.

Reference 2 - 0.95% Coverage

Your mood suddenly changes when you realized that you have physical limitations.

Reference 3 - 0.34% Coverage

I want to be alone and sleep.

Reference 4 - 1.18% Coverage

I require silence; I don't want to be disturbed by any noise, not even by the voices of my children.

Reference 5 - 1.37% Coverage

You might feel depressed if your friends leave you simply because you are empty; it is embarrassing and uncomfortable

<Files\\Data\\P07> - § 3 references coded [3.10% Coverage]

Reference 1 - 0.80% Coverage

Now, sometimes I have a squeezing pain in my left chest, and I feel tired.

Reference 2 - 1.77% Coverage

When medicines disappeared, I feel stressed. I feel like my symptoms would be aggravated. You know…. in that situation, you worry a lot about getting the medicine.

Reference 3 - 0.52% Coverage

I feel depressed because of my physical limits.

<Files\\Data\\P08> - § 5 references coded [3.86% Coverage]

Reference 1 - 1.37% Coverage

I feel tired when I'm working, I can't work hard, I can't lift weight, sometimes I feel like I’m losing my consciousness

Reference 2 - 0.22% Coverage

I am very depressed

Reference 3 - 1.14% Coverage

I don't have what I want, this makes me to feel hopeless and disappointed. This has hurt me so much.

Reference 4 - 0.42% Coverage

I like to be alone and pray at church

Reference 5 - 0.70% Coverage

You get anxiety when you think your life before this illness.

<Files\\Data\\P10> - § 4 references coded [1.51% Coverage]

Reference 1 - 0.74% Coverage

I have trouble breathing when I speak and sleep

Reference 2 - 0.20% Coverage

I'm depressed

Reference 3 - 0.22% Coverage

worry and fear

Reference 4 - 0.35% Coverage

depression and anxiety

<Files\\Data\\P11> - § 2 references coded [1.48% Coverage]

Reference 1 - 0.58% Coverage

pecially after long and uphill walks.

Reference 2 - 0.90% Coverage

I am frustrated having to take medications all the time.

<Files\\Data\\P12> - § 3 references coded [3.74% Coverage]

Reference 1 - 1.64% Coverage

I have chest pain and I feel tired while I'm walking uphill. I become fatigued as well while I am working hard.

Reference 2 - 0.70% Coverage

I worry that one day my heart may stop beating.

Reference 3 - 1.40% Coverage

I fear going to sleep because I feel like I'm going to die. This entire sensation depresses me.

<Files\\Data\\P13> - § 5 references coded [11.39% Coverage]

Reference 1 - 3.28% Coverage

Even though my symptoms significantly improved after starting the medications, I still experience fatigue. I have oedematous feet and am experiencing numbness. Moreover, I experience chest pain and palpitations.

Reference 2 - 0.69% Coverage

I feel down when I forget to take my pills.

Reference 3 - 1.17% Coverage

You will be depressed when you realized that you need someone to support you

Reference 4 - 1.59% Coverage

My condition prevented me from working and made me dependent. All of this is quite mentally unsettling.

Reference 5 - 4.66% Coverage

I often forget to take my pills. I feel stupid when I forget it. I mean, why I forget my life. In that case, my sister reminds me to take it. She always asks me whether I taken my pills. I think you will feel depressed when your symptoms are getting worse and worse because of forgetting your pills.

<Files\\Data\\P14> - § 2 references coded [2.41% Coverage]

Reference 1 - 1.19% Coverage

I feel tired and I have difficulty of going upstairs. I get fatigue easily.

Reference 2 - 1.22% Coverage

At first, I felt depressed, I thought a lot about this why this happened to me.

<Files\\Data\\P01> - § 10 references coded [16.38% Coverage]

Reference 1 - 1.15% Coverage

I feel difficulty of breathing when i try to do a little activity like walking.

Reference 2 - 1.74% Coverage

I have less energy to perform strenuous exercises, go for walks in the hot environment, and engage in other activities.

Reference 3 - 1.72% Coverage

I used to go to funerals and visit people who were ill and hospitalised, but I have stopped doing all of these things

Reference 4 - 0.64% Coverage

I have restrictions on my physical activity.

Reference 5 - 1.26% Coverage

This disease wants you to have rest, and I think it is the diseases of rich people.

Reference 6 - 1.27% Coverage

Heart failure is horrible. I am unable to perform daily activities without difficulty.

Reference 7 - 1.50% Coverage

I become additional burden on my family because i need their close support during my daily activities.

Reference 8 - 3.89% Coverage

Heart failure have a great impact on work as it has a great limitation in physical activities, so that you can’t work as before. Before this disease, I worked in several types of jobs but now I stopped so I do have no income now which could affect my living status.

Reference 9 - 1.11% Coverage

if there is something worrying, that could affect my sleep during the night.

Reference 10 - 2.09% Coverage

I totally stopped social activities and relationships because I can’t easily travel to attend and actively participate in social activities.

<Files\\Data\\P02> - § 3 references coded [6.80% Coverage]

Reference 1 - 2.42% Coverage

I'm currently limited in what I can do because I can't walk as fast as I used to. I was quite quick. I'm moving slowly as a result.

Reference 2 - 1.74% Coverage

Heart failure affects work since it restricts activity and prevents you from doing heavy work.

Reference 3 - 2.64% Coverage

Due to your inability to actively participate and your restrictions on movement, you will become isolated from social events and relationships.

<Files\\Data\\P03> - § 3 references coded [8.51% Coverage]

Reference 1 - 1.99% Coverage

there was time for me to have difficulty of going to bathroom and I was not able to control myself; It makes me feel depressed why am not doing things as I used to be.

Reference 2 - 3.61% Coverage

At work, my co-workers frustrate with my case more than I do. They feel that people with heart failure are more vulnerable to sudden death. For instance, if you need to borrow money, nobody wants to take the risk by giving it to you because they worry that you might die suddenly before paying them back.

Reference 3 - 2.92% Coverage

when I visit my friends, they frequently ask about how I'm feeling and ask, "How do you do?", How do you feel? I always respond that I'm OK. I know that I am taking my medication as directed and feeling better, yet their questions are disturbing.

<Files\\Data\\P04> - § 16 references coded [22.92% Coverage]

Reference 1 - 0.49% Coverage

I can’t walk for long distance.

Reference 2 - 0.70% Coverage

I have inability to lift and carry heavy object.

Reference 3 - 1.21% Coverage

I might seem in good physical condition, but my body is weak, which depresses me.

Reference 4 - 0.29% Coverage

I can't do heavy job

Reference 5 - 0.49% Coverage

I can't use a hammer to fix things

Reference 6 - 1.57% Coverage

I move slowly when working since I can't do it quickly. My boss regularly criticised me for moving slowly.

Reference 7 - 2.74% Coverage

I feel down when I feel tired of doing ordinary activities at home…when I feel depressed I have nothing to do ..just nothing, I just sit down and take a rest and there's no other solution.

Reference 8 - 1.57% Coverage

My life right now is very miserable. I'm angry that I can't go out and get in as I want. This is the worst.

Reference 9 - 2.30% Coverage

I used to chew khat with my friends but am not doing that so they don’t want me; even I don’t have money to enjoy with them; so I am isolated from my friends.

Reference 10 - 1.66% Coverage

My co-workers work other jobs in their free time and earn extra money, but I am unable to do so due to my health.

Reference 11 - 1.43% Coverage

I get conflict with my wife since my heart failure has made it difficult to have sex with my wife.

References 12-13 - 3.26% Coverage

I wake up at night due to chest pain, which worries me. I was socially active before but now I stopped all social activities and relationships because I'm afraid of what would be if something bad happened to me on the street

Reference 14 - 0.80% Coverage

My families are always depressed because of my illness.

Reference 15 - 1.64% Coverage

I don't eat what I want. I become upset that I don't eat something delicious when I see someone else enjoying it.

Reference 16 - 2.76% Coverage

It's hard for me to stand up and bow down at mosque. During one mosque visit, I observed an elderly woman bowing down easily despite being older than I am. I feel like am dead; why not me?

<Files\\Data\\P05> - § 3 references coded [5.17% Coverage]

Reference 1 - 0.64% Coverage

I'm too exhausted to do hard jobs.

Reference 2 - 1.80% Coverage

My income is affected because I used to work as much as I wanted but now, I don't work as hard.

Reference 3 - 2.73% Coverage

I can't enjoy myself with my friends. Since I'm weak and have no income, compared to my friends who work and make money, I feel quite emotional

<Files\\Data\\P06> - § 12 references coded [16.22% Coverage]

Reference 1 - 0.36% Coverage

I want rest and emotional calm.

Reference 2 - 0.50% Coverage

I stopped working after having this illness

Reference 3 - 1.22% Coverage

If I were healthy, I wouldn't have to quit my job, but because of this sickness, everything has changed.

Reference 4 - 1.06% Coverage

I don’t have the capacity to do everything, and I am performing limited activities slowly.

Reference 5 - 0.82% Coverage

I can't even visit a market alone because I can’t lift anything heavy

Reference 6 - 1.72% Coverage

I have trouble breathing properly, and I have trouble falling asleep at night. However, I have improvement after I started my medical care here.

Reference 7 - 2.17% Coverage

I feel upset when people turned on you as a result of my current condition. As I currently don't have any money, many people may ignore me because they associate friendship with money.

References 8-9 - 3.82% Coverage

I've been good in social life before and I'm involved in anything in social activities. I was close to helping and consulting someone. But I stopped everything after this condition, am not even visiting when someone is very sick and dead. I don't have any relationship with friends and relatives, and now I'm not going anywhere

Reference 10 - 1.62% Coverage

I recently attempted to attend a funeral and went to do so, but the weather was quite hot, so I turned back and walked home with support.

Reference 11 - 1.62% Coverage

I got this illness when I was at my most productive age and am now unable to engage in physical activities, which makes me feel depressed.

Reference 12 - 1.30% Coverage

My income is greatly impacted by how much money I make; we used to have a good life, but now there is nothing.

<Files\\Data\\P07> - § 10 references coded [17.82% Coverage]

Reference 1 - 1.95% Coverage

I used to actively engage in social activities, visit sick people, and have fun with my friends. Now, due to my illness, I am no longer as active in friendships and relationships.

Reference 2 - 2.49% Coverage

I have difficulty to carry out my previous activities, such as gardening at my home and providing for them with care. I find it difficult to pick up items and move them. I am unable to walk for long distance, if so, I feel tired.

Reference 3 - 2.68% Coverage

I used to be a big fan of social relationships when I was healthy. I used to go to my relatives on holidays, but now I stopped that. I would love to relax with my friends, but I can’t do that since I have several limitations to have fun with them.

Reference 4 - 2.06% Coverage

I won't participate in social activities because people will push you to consume alcohol and fatty foods, and they won't understand if you don't want to eat and drink. This has a big impact.

Reference 5 - 1.37% Coverage

I have HTN, DM and HF. So, I have a lot of things to think and worry, which makes me to have trouble sleeping during the night

References 6-7 - 3.23% Coverage

This condition has a significant impact on your job since it makes it difficult to move around and prevents you from working because you have physical limitations or a failing heart. Your income will be significantly impacted if you are not working. As a result, life will get challenging for you.

References 8-9 - 2.54% Coverage

Sleeping is difficult, especially when experiencing symptoms. You won't be able to sleep if the chest pain is severe; you'll also be disturbed and anxious. Your family feels horribly anxious alongside you, which is difficult for them.

Reference 10 - 1.50% Coverage

People may isolate you, for example, when you cough, they may treat you differently and think you have COVID 19. You find this upsetting.

<Files\\Data\\P08> - § 13 references coded [30.66% Coverage]

Reference 1 - 1.56% Coverage

I can't walk far, and I can't finish what I start. It is even hard for me to came from home to this hospital for my regular follow up.

References 2-3 - 3.32% Coverage

Now, I have complete loss of income since am not working due to this illness. I never worried about my living expenses before my illness, but now, it's difficult to pay for basic necessities. My wife is attempting to make payments on the expenses. So that I feel like I am a burden on her.

Reference 4 - 3.78% Coverage

This illness has also had an impact on my marriage because I feel less completely motivated to engage in sexual activities. I used to have a good sexual relationship with my wife, but now it has impaired my entire sexual desire. My wife thinks that I am dating another woman because of my seeming lack of interest in intimacy.

Reference 5 - 2.53% Coverage

I used to work hard, but now I struggle to complete anything. I used to be a taxi driver and would work up to 16 hours a day; now, if I don't get enough sleep, I wake up feeling fatigued and am unable to do my job duties.

Reference 6 - 0.76% Coverage

I am very depressed because I can't meet the needs of my children.

Reference 7 - 1.89% Coverage

Because of my cardiac condition, I can't work, I can't travel far to get what I want, and I can't cope with high temperatures, so I spend a lot of time sitting down.

Reference 8 - 1.47% Coverage

I am now unemployed and dependent on my wife. My wife is taking care of everything now…that is depressive to be dependent on her

Reference 9 - 1.12% Coverage

It has some influence on sleep, especially when am anxious; that I don't sleep until the morning.

References 10-11 - 6.55% Coverage

I already isolated from social activities and relationship. For instance, even if someone invites me to a party, I won't go since I know I won't be able to dance with the other people there because of my illness; if I did, I might fall over and disturb the party. When you go to do something, and you realize that you can’t do it. For instance, one day I was walking fast to take the bus. I see 70-year-old women passed me for a bus but am late to reach the bus, it leaves. At that moment I really feel like I am worthless. I become panic to why this is happening to me.

Reference 12 - 7.03% Coverage

For example, one day one of my neighbours was died, I can't actively participate like others to facilitate the burial, I simply watched what others are doing, in that case people do not understand me. They said “we are working but you are simply sitting and watching, why don’t you help us “. They were disappointed by my inactivity. Then, I went and helped them; I worked a little hard and fell in the middle of them. Later, with that feeling, I went to the

funeral. Then, I felt fatigued because it was so far away and the weather was so hot. Then I fell again at the cemetery, and people carried me to my home.

Reference 13 - 0.66% Coverage

you can't do what others are doing, you have limitations.

<Files\\Data\\P09> - § 4 references coded [12.27% Coverage]

Reference 1 - 4.22% Coverage

I am unable to perform ordinary activities—such as cooking, cleaning, dish washing, and baking bread. There is nothing I can do now. It is embarrassing.

Reference 2 - 1.97% Coverage

You find it difficult to walk for long distances, you get tired easily.

Reference 3 - 3.33% Coverage

I couldn't walk a long distance. I need someone to support me even to come in this hospital for my monthly appointment.

Reference 4 - 2.75% Coverage

I'm not visiting a church. I am not fasting because of my meds, which has an impact on my religion.

<Files\\Data\\P10> - § 10 references coded [27.82% Coverage]

Reference 1 - 2.25% Coverage

I used to attend to church and pray, but I am unable to do so anymore since I stay at home all the time, feel exhausted, and am unable to go.

Reference 2 - 1.65% Coverage

I cannot fast since I must take my medication in the morning; this interferes with my religious practice.

Reference 3 - 7.68% Coverage

I asked God if my time is over, I don’t want to suffer any more and am not scared of dying, let me go. The fatigue is overwhelming; I can't even do simple tasks without difficulty. It's been really challenging. I am exhausted with everything, and I feel like my health is getting worse and worse. I am not a happy person now because I am not feeling good. I feel really dizzy, fatigue and exhausted. I never thought I would have to go all this…….all I can do is accepting my condition.

Reference 4 - 1.73% Coverage

I used to be able to work without any limitations, but now I'm too fatigued to do anything. I can't even walk.

Reference 5 - 1.65% Coverage

I am exhausted, fatigue and not sleeping very well during the night due worry and fear of my condition.

Reference 6 - 0.91% Coverage

My depression and anxiety cause me to be awake at night.

Reference 7 - 3.23% Coverage

It has an impact on family. I was able to come in this hospital alone but now I can’t. My daughter reduced some of her working hrs to bring me here every month and she support me with all of my activities.

Reference 8 - 2.44% Coverage

My friends and I used to hang out and have fun, but after I got sick, they all stopped coming to see me. They don’t want me since I am dependent on others.

References 9-10 - 6.27% Coverage

I'm having trouble sleeping and I suddenly woke up in the night because I don't feel psychologically well, worry a lot about my health, need for medication, and dependence on my daughter. Due to my condition, I am not

as involved in social activities and relationships as I once was. Of course, my neighbours try to understand me because the knew my current condition, but I feel socially isolated.

<Files\\Data\\P11> - § 4 references coded [14.72% Coverage]

Reference 1 - 2.80% Coverage

You know I am not working hard and earn money as my friends; some of my friends have extra jobs and earn lots of money. But I have limitations so I can’t work as much as I want.

Reference 2 - 3.14% Coverage

I'm sick and always taking medicine. How much longer will my life be like this? Sometimes I asked God why this happening on me? and then I become calm and put my faith in God and let him handle it.

Reference 3 - 2.28% Coverage

I have difficulty of walking long distance at speed. I find it challenging to go shopping because I can only lift and carry up to 5 kilograms.

Reference 4 - 6.49% Coverage

My family is impacted by this problem because they are the ones that help me with my medical treatment. They take care of me, and I value having them in my life. They don't want to know that I'm sick. I try not to worry my family. They worry a lot when I'm ill. They always called me to check me; they just feel that you are fragile. Wow, they are very helpful …I don’t want to imagine my life without my family.

<Files\\Data\\P12> - § 8 references coded [16.17% Coverage]

Reference 1 - 2.50% Coverage

I worry whenever I consider travelling because I am unable to walk long distances and my home is somewhat steep. Am worried about what might happen if I got sick somewhere.

Reference 2 - 1.53% Coverage

When your physical ability isn't as good as it used to be, you feel incompetent, which is embarrassing.

Reference 3 - 1.24% Coverage

I have difficulty of doing simple tasks such as cooking, washing dishes and cleaning.

Reference 4 - 1.06% Coverage

I am not going for shopping since am unable to carry heavy shopping bags.

Reference 5 - 1.28% Coverage

If you're employed and you don't do the job properly, there's also a loss of that job.

Reference 6 - 3.00% Coverage

Since my illness, I already isolated from my friends because most of them live far from me, and I can no longer enjoy my time with them as much as I used to due to my limitations and financial difficulties.

Reference 7 - 3.81% Coverage

Because you desire to work but are physically limited, this sickness has a significant impact on your ability to do so. If you are unable to work, your income will decrease. I'm frustrated of not working as much as I used to and not having enough money to live.

Reference 8 - 1.75% Coverage

I used to be engaged in social activities and relationships, but due to my sickness, I no longer actively participate.

<Files\\Data\\P13> - § 8 references coded [21.49% Coverage]

Reference 1 - 2.33% Coverage

Due to my condition, I am currently unemployed, which is embarrassing. I used to work really hard, make a lot of money, and even provide for my family.

Reference 2 - 1.96% Coverage

I used to be able to work without any limitations, but now I'm too fatigued and I don't have the energy to do things as before.

Reference 3 - 2.65% Coverage

You will be depressed when you realized that you need someone to support you. I feel like am dependent on my family. When I feel down, I pray; when I pray, I feel better.

Reference 4 - 1.91% Coverage

The family is affected because you become dependent on your family. I sometimes wonder what would happen if I had no family.

Reference 5 - 3.89% Coverage

I am not meeting my friends as before; I stop to meet friends due to my restrictions. I feel like am not competent like my friends. So, I can't have fun with them because of my disease and I don't want to deal with fatigue that makes my symptoms worse.

Reference 6 - 3.25% Coverage

I used to be good at social activities and relationships but now I'm down and the disease limit your physical activities. I can't come close to participate in social activities, so I just rather not get started.

Reference 7 - 1.77% Coverage

It's challenging to be dependent on someone while not working. Am unable to even work and pay for my prescriptions.

Reference 8 - 3.73% Coverage

When people look at me, they think that I am healthy, but I have health issue. I am just 43 and I said why this happened to me at my productive age, am still young. If I were like 60 or 70, I would be accepting this illness. This is horrible.

<Files\\Data\\P14> - § 8 references coded [23.98% Coverage]

Reference 1 - 1.25% Coverage

My condition prevents me from working as before, so I reduced on my working hours

Reference 2 - 3.59% Coverage

I have physical limitations for instance, I have difficulty of walking long distance at speed. I have to sit down and rest…I have to walk at slower pace. This affected my daily life since am not active as I was before this illness.

Reference 3 - 2.89% Coverage

I can't be as physically active as I once could because of my limitations. I'm unable to carry my kids very far and I can't even play with them like I used to. I can’t walk long distance.

Reference 4 - 1.04% Coverage

I used to go visiting friends and relatives but now I stopped that.

Reference 5 - 3.44% Coverage

I have stopped hanging out with my friends because when they are doing something fun…like drinking alcohol, I am going to be “No”…I don’t want really…. so, I am not meeting them. At least I have to survive for my children.

Reference 6 - 4.63% Coverage

I am a taxi driver so after this illness I reduced my working hours since I have difficulty of going out in hot weather. Of course, it affects income…it doesn’t allow fully to be back to work. If you are not working, you will have no adequate income for family living expenses, which is frustrating.

Reference 7 - 5.28% Coverage

My inability to work affects me a lot. When I start working, you know….I feel fatigue and tired. I can't work the whole day, so I get frustrated with not able to work and have the energy as before. So, I am not working as before because of my illness, so it affects my income. In this case, you feel dissatisfied by your life and feel down.

Reference 8 - 1.86% Coverage

My brothers have been supportive, you know, they really helped me, of course being dependent on other is also stressful.

<Files\\Data\\P01> - § 1 reference coded [1.83% Coverage]

Reference 1 - 1.83% Coverage

having a full-earned income and being happy and healthy without any illness and without out any difficulty of doing things.

<Files\\Data\\P02> - § 1 reference coded [1.28% Coverage]

Reference 1 - 1.28% Coverage

being physically active, eating and drinking without any restriction.

<Files\\Data\\P03> - § 1 reference coded [1.42% Coverage]

Reference 1 - 1.42% Coverage

Health-related quality of life means the physical ability to do what you want and being physically healthy, no illness.

<Files\\Data\\P04> - § 1 reference coded [1.53% Coverage]

Reference 1 - 1.53% Coverage

Health-related quality of life means being happy, work with out any limitation and have enough income.

<Files\\Data\\P05> - § 1 reference coded [1.69% Coverage]

Reference 1 - 1.69% Coverage

staying healthy and having adequate income to cover basic needs like food and house rent.

<Files\\Data\\P06> - § 1 reference coded [1.20% Coverage]

Reference 1 - 1.20% Coverage

Health-related quality of life means being healthy means that if you have health, you can get anything.

<Files\\Data\\P07> - § 1 reference coded [2.13% Coverage]

Reference 1 - 2.13% Coverage

I think staying healthy and financially secure are key components of health-related quality of life. If I have adequate income, I could be happy and comfortable with my life…. I think that is QoL.

<Files\\Data\\P08> - § 1 reference coded [0.33% Coverage]

Reference 1 - 0.33% Coverage

living a healthier lifestyle.

<Files\\Data\\P09> - § 1 reference coded [1.47% Coverage]

Reference 1 - 1.47% Coverage

Health-related quality of life means a healthier life

<Files\\Data\\P11> - § 1 reference coded [1.47% Coverage]

Reference 1 - 1.47% Coverage

Health-related quality of life means being healthy and efficient physically and economically.

<Files\\Data\\P12> - § 1 reference coded [1.43% Coverage]

Reference 1 - 1.43% Coverage

Health-related quality of life means being fully healthy and living without physical limitations.

<Files\\Data\\P13> - § 1 reference coded [1.34% Coverage]

Reference 1 - 1.34% Coverage

Health-related quality of life means being healthy, happy, and live without anxiety.

<Files\\Data\\P14> - § 1 reference coded [1.35% Coverage]

Reference 1 - 1.35% Coverage

Health-related quality of life means having enough income and live without any stress.

<Files\\Data\\P01> - § 10 references coded [10.91% Coverage]

Reference 1 - 3.87% Coverage

I'm feeling good when I'm relaxing, and when I'm doing a little exercise, it's a problem. I feel exhausted while am trying to do some ordinary activities at home, that makes me frustrated, but I feel good after a brief rest. Resting makes me a little more relaxed.

Reference 2 - 0.29% Coverage

regular HF follow-up

Reference 3 - 1.20% Coverage

I play and talk with my family members so that I can forget about my depression.

Reference 4 - 0.58% Coverage

I attend monthly follow-up appointments

Reference 5 - 0.48% Coverage

take my medications as prescribed

Reference 6 - 0.92% Coverage

My family are there for me; without them, I could not survive.

Reference 7 - 0.79% Coverage

I changed my diet to avoid fatty meals and reduce salt

Reference 8 - 0.61% Coverage

I watch television and play with my family

Reference 9 - 1.21% Coverage

I always pray by saying “Dear God, please bless me with good health and strength”.

Reference 10 - 0.95% Coverage

I've always tried to feel happy, and I don't want to feel down.

<Files\\Data\\P02> - § 14 references coded [15.66% Coverage]

Reference 1 - 1.00% Coverage

When I rest and take some rosemary tea, I feel better.

Reference 2 - 0.63% Coverage

I receive regular check-up monthly

Reference 3 - 0.70% Coverage

I also take herbal products (rosemary)

Reference 4 - 0.20% Coverage

holy water.

Reference 5 - 1.87% Coverage

I have accepted this condition and am waiting for the day when I will no longer be able to survive.

Reference 6 - 1.26% Coverage

I slept when I was feeling down; when I woke up, I feel refreshed.

Reference 7 - 0.67% Coverage

I feel better when I take anti pain.

Reference 8 - 2.72% Coverage

I'm talking to a friend about it; we talked finding better medical care and sharing our illnesses and feelings. I feel better as a result of this.

Reference 9 - 1.48% Coverage

I'm grateful for my family because my wife watches what I eat very carefully.

References 10-11 - 2.55% Coverage

I exercise physically through walking, which is incredibly beneficial. I changed my diet by staying away from fatty meals and excess salt.

Reference 12 - 0.52% Coverage

I stopped consuming alcohol.

Reference 13 - 1.46% Coverage

I feel better after consuming a cap of rosemary tea rather than the medication.

Reference 14 - 0.61% Coverage

take my medication as prescribed.

<Files\\Data\\P03> - § 9 references coded [7.08% Coverage]

Reference 1 - 0.86% Coverage

I've had regular heart failure and diabetes treatment follow up and care.

Reference 2 - 0.91% Coverage

When I feel tired, I sit down and take a rest. This makes me to feel better.

Reference 3 - 0.50% Coverage

I am correctly adhering to my medications;

Reference 4 - 0.71% Coverage

I have a monthly appointment and have regular medical checks

Reference 5 - 0.79% Coverage

I modify my diet and avoid fatty foods and consuming low salt diet.

Reference 6 - 1.00% Coverage

engaged in some regular simple physical exercise like walking for 30 minute each day.

Reference 7 - 0.76% Coverage

I try to stay calm even when I am feeling tired to avoid stress.

Reference 8 - 0.48% Coverage

I enjoyed and relaxed by watching movies.

Reference 9 - 1.06% Coverage

I used to smoke cigarettes and drink alcohol, but I've stopped doing both of those things.

<Files\\Data\\P04> - § 5 references coded [3.90% Coverage]

Reference 1 - 0.22% Coverage

I take medicine

Reference 2 - 1.22% Coverage

I reduced the amount of salt in my diet and stopped taking alcohol and fatty meals.

Reference 3 - 0.26% Coverage

I modified my diet

Reference 4 - 0.67% Coverage

am having regular appointment with physicians

Reference 5 - 1.53% Coverage

I used to drink alcohol, smoke cigarettes and chewing khat, but now I stopped doing all of those things.

<Files\\Data\\P05> - § 6 references coded [4.96% Coverage]

Reference 1 - 0.64% Coverage

I feel better when I take a rest.

Reference 2 - 0.30% Coverage

all I do is pray

Reference 3 - 0.47% Coverage

I didn't work very hard.

Reference 4 - 1.02% Coverage

I don't always lose my faith; I go to church and pray.

References 5-6 - 2.52% Coverage

I follow my medications and perform my doctors' advice. I am coping with the help of "Mother Teresa Missionaries of Charity club".

<Files\\Data\\P06> - § 10 references coded [9.43% Coverage]

Reference 1 - 0.47% Coverage

When I sit down and rest, I feel better.

Reference 2 - 0.90% Coverage

I am having regular monthly follow up at the cardiac clinic of this hospital

Reference 3 - 0.36% Coverage

I am also using herbal products

Reference 4 - 0.14% Coverage

holly water.

Reference 5 - 0.82% Coverage

I have learnt to live with my condition and wait for the mercy of God

Reference 6 - 4.53% Coverage

When you are dealing with an illness like heart failure, social support is crucial. My husband and children are helping me a lot, I feel comfortable. My brothers who live abroad also provide me with support, assisting me to manage my health and sometimes sending me money and medications. Their support has been crucial to my survival, and I would find it difficult to live without them.

Reference 7 - 1.33% Coverage

I'm learning to accept everything now. If you leave things up and go back to yourself, anything is easy to adjust.

Reference 8 - 0.32% Coverage

I follow my doctor's advice

Reference 9 - 0.15% Coverage

I always pray

Reference 10 - 0.41% Coverage

I take my medications as prescribed

<Files\\Data\\P07> - § 9 references coded [7.10% Coverage]

Reference 1 - 0.55% Coverage

My symptoms reduced after start taking medications

Reference 2 - 0.68% Coverage

I now have a medical check-up on my heart failure every month.

Reference 3 - 0.99% Coverage

I monitor what I eat; I avoid salt; and I use sunflower oil instead of eating fatty foods.

Reference 4 - 0.78% Coverage

I accepted my condition and following the instructions from the doctors.

Reference 5 - 1.85% Coverage

It is the matter of accepting your condition. If you are not accepting, you will be depressed. Although I have HF, this does not necessarily mean that I am going to die.

References 6-7 - 1.04% Coverage

reducing salt and avoid fatty foods. I am doing regular physical exercise. I monitor my weight,

Reference 8 - 0.51% Coverage

I am following my routing monthly appointments.

Reference 9 - 0.68% Coverage

I am taking care of myself, and I try to forget my condition.

<Files\\Data\\P08> - § 6 references coded [5.48% Coverage]

Reference 1 - 0.79% Coverage

I pray to God and use holy water, both of which are quite beneficial.

Reference 2 - 0.98% Coverage

I believe there will be a day of God's mercy when I can overcome all of this trouble.

Reference 3 - 1.24% Coverage

I like to be alone and pray at church when I'm feeling down. Then, once I'm feeling better, I return home.

Reference 4 - 1.85% Coverage

I've avoided things that are uncomfortable for the illness, such as avoiding fatty foods; avoiding alcohol; avoiding addictive substances. I am taking vegetables.

References 5-6 - 0.62% Coverage

I monitor my weight. I'm taking my medicines properly.

<Files\\Data\\P09> - § 5 references coded [7.50% Coverage]

Reference 1 - 2.39% Coverage

I attend the outpatient department on a monthly, and I take the prescribed medications

Reference 2 - 1.64% Coverage

After I started taking the drug, my symptoms have improved.

Reference 3 - 1.47% Coverage

I use holy water in addition to prescription drugs.

Reference 4 - 0.72% Coverage

I sleep when I feel down.

Reference 5 - 1.28% Coverage

I'm taking my medicines properly as prescribed

<Files\\Data\\P10> - § 6 references coded [8.00% Coverage]

Reference 1 - 1.10% Coverage

I put two pillows in my back to drop myself so I can breathe better.

References 2-3 - 3.23% Coverage

I sometimes visit for regular cardiac follow-up every two weeks, and other times every month. I have started taking a prescription for heart failure in addition to my diabetes and high blood pressure meds,

Reference 4 - 2.41% Coverage

I must struggle for as long as God wills; I am thankful and believe that everything has happened for the good. I don't criticise God for giving it to me.

References 5-6 - 1.26% Coverage

I am coping with my God. I am also taking medicine appropriately as prescribed.

<Files\\Data\\P11> - § 11 references coded [8.95% Coverage]

Reference 1 - 0.79% Coverage

I feel better while I'm reading books and working.

Reference 2 - 1.88% Coverage

I am taking five different types of medications as prescribed and have regular monthly follow-ups for my heart failure.

Reference 3 - 0.80% Coverage

I frequently read books to relax when I'm stressed.

Reference 4 - 0.27% Coverage

praying in church

Reference 5 - 1.75% Coverage

I try to forget what has happened to me and I try to keep my mind off from things that makes me to feel down.

Reference 6 - 0.90% Coverage

I take a break when I start to feel tired while working.

Reference 7 - 0.61% Coverage

I take my prescription meds as advised.

Reference 8 - 0.66% Coverage

avoiding heavy work, avoiding hot weather,

Reference 9 - 0.33% Coverage

monitoring my weight,

Reference 10 - 0.33% Coverage

avoiding fatty foods.

Reference 11 - 0.63% Coverage

I go to church every day and I pry quiet

<Files\\Data\\P12> - § 8 references coded [7.32% Coverage]

Reference 1 - 1.32% Coverage

I have regular monthly follow-up visit in this hospital and take medications as prescribed.

Reference 2 - 0.63% Coverage

I also use holy water along with my meds.

Reference 3 - 0.97% Coverage

shared ideas with other people while I was here for my appointment

Reference 4 - 2.07% Coverage

We shared strategies for dealing with our symptoms and other problems, which helped to reduce my anxiety and depression and made me feel good.

Reference 5 - 0.51% Coverage

I tried to relax and avoid stress.

Reference 6 - 0.54% Coverage

stay away from salty and fatty foods.

Reference 7 - 0.60% Coverage

I also followed the advice of the doctor.

Reference 8 - 0.68% Coverage

Even though I have symptoms, I try to feel calm

<Files\\Data\\P13> - § 7 references coded [8.40% Coverage]

Reference 1 - 0.43% Coverage

When I pray, I feel better.

Reference 2 - 1.11% Coverage

I have monthly appointment at cardiac clinic and am taking medications.

Reference 3 - 0.32% Coverage

I also prayed to God

Reference 4 - 0.89% Coverage

spiritual counselling and bible learning and meditation.

Reference 5 - 2.17% Coverage

I had come to understand that this illness is a gift from God. I thus decided to live with it in the hopes that God will make things right.

Reference 6 - 2.94% Coverage

I am coping with the help my God. I feel I have a good relationship with God. I am always prying and reading bible. I might not be able to do things I used to do but I believe God is with me.

Reference 7 - 0.52% Coverage

taking my medicine as prescribed.

<Files\\Data\\P14> - § 14 references coded [12.34% Coverage]

Reference 1 - 0.63% Coverage

I've had monthly heart failure check-ups

Reference 2 - 0.60% Coverage

I should avoid alcohol and salty foods.

Reference 3 - 0.25% Coverage

I pray to God.

Reference 4 - 0.59% Coverage

I spend time with my family at home.

Reference 5 - 1.58% Coverage

I've reconciled with my condition. I had to start by accepting it in to prevent it from getting worse.

Reference 6 - 0.60% Coverage

I'm taking my medications as prescribed

Reference 7 - 1.08% Coverage

I've reduced salt. I avoided fatty foods, and I mostly eat vegetables.

Reference 8 - 0.37% Coverage

I don't consume alcohol,

Reference 9 - 0.93% Coverage

I exercise regularly. I am working on getting my weight down

Reference 10 - 1.46% Coverage

I am keeping track of my weight, which I dropped from 90 to 80 kg. I want to lose more weight.

Reference 11 - 2.32% Coverage

Also try to collect information from other patients about their experience regarding the management of this condition especially dietary modification.

Reference 12 - 0.68% Coverage

I stay calm even when I am not feeling good.

Reference 13 - 0.63% Coverage

I take my mind off it by watching movies.

Reference 14 - 0.60% Coverage

I just do something that takes my mind.

<Files\\Data\\P01> - § 1 reference coded [1.40% Coverage]

Reference 1 - 1.40% Coverage

I am unable to see the same doctor each month. In each appointment, I found a different doctor.

<Files\\Data\\P02> - § 2 references coded [12.17% Coverage]

Reference 1 - 2.39% Coverage

Due to the high cost of healthcare in private hospitals, I have taken some medication and have chosen to begin my follow-up here.

Reference 2 - 9.78% Coverage

when I arrived for my appointment, the physicians occasionally changed. I've never gone to the same physician. As a result of the physician's poor handwriting, I have observed them becoming confused about my most recent history when reading my chart. Their colleague's handwriting was difficult for them to read. So, they just continue to prescribe the same drug. They respond to you fast, don't fully consider my complaints, and just want to get it over with so they can go on to the next patient. This is really embarrassing.

<Files\\Data\\P03> - § 4 references coded [6.51% Coverage]

Reference 1 - 1.84% Coverage

I bought enough medication for two months because I knew that medications can suddenly disappear from the country. The cost of medicines is relatively high.

Reference 2 - 0.76% Coverage

I get confused when I can’t find the medicine from the pharmacy.

Reference 3 - 0.60% Coverage

Anyone who can't afford that is sentenced to death.

Reference 4 - 3.31% Coverage

The government also focuses on infectious diseases such as AIDS and TB, and I think the medication is free. these people are living normal lives getting to benefit. People with non-communicable diseases, however, often die because they are unable to afford for their medication.

<Files\\Data\\P04> - § 1 reference coded [1.28% Coverage]

Reference 1 - 1.28% Coverage

I have additional gastritis and am asthmatic, so I have extra expenses for medications.

<Files\\Data\\P05> - § 1 reference coded [1.23% Coverage]

Reference 1 - 1.23% Coverage

My symptoms get worse when I have no money to cover my house rent

<Files\\Data\\P07> - § 2 references coded [2.42% Coverage]

Reference 1 - 0.65% Coverage

The worst is when a medication disappears off the market.

Reference 2 - 1.77% Coverage

When medicines disappeared, I feel stressed. I feel like my symptoms would be aggravated. You know…. in that situation, you worry a lot about getting the medicine.

<Files\\Data\\P08> - § 3 references coded [9.90% Coverage]

Reference 1 - 2.98% Coverage

Since coming here, I've started taking medications and have regular appointments every month. However, no doctor has ever examined me, and I once gave a blood sample, but the results haven't been returned. I continue to come here simply to take my medications.

Reference 2 - 4.41% Coverage

I have never had the same doctor when I come during my appointment. It is always a new one. The doctors have not got enough time for you. I personally expect doctors to provide adequate information about my progress. They asked quickly and give your prescription. Consequently, I understand that I'm not receiving better medical care here, which is disturbing and affected my progress.

Reference 3 - 2.52% Coverage

purchasing medicines on the open market is difficult and costly. I buy what I need if I can afford it; however, if not, I will not buy that medication. In that case, missing that medication will make my symptoms worse.

<Files\\Data\\P09> - § 1 reference coded [3.47% Coverage]

Reference 1 - 3.47% Coverage

You might find it difficult to get the medicines your doctor has prescribed for you; it may be disappeared from the market.

<Files\\Data\\P10> - § 2 references coded [3.04% Coverage]

Reference 1 - 0.91% Coverage

Medicine is expensive and hard to find on the open market.

Reference 2 - 2.13% Coverage

I thought I was going to pass away when the medication was taken off the market. You can’t get the medicine easily and it is expensive.

<Files\\Data\\P11> - § 2 references coded [2.44% Coverage]

References 1-2 - 2.44% Coverage

The cost of medications is very high and finding them is difficult. I came to this hospital because medical expenses are high in private hospitals as well.

<Files\\Data\\P12> - § 1 reference coded [3.74% Coverage]

Reference 1 - 3.74% Coverage

The expenses related to managing heart failure is high, and there are several costs involved, including those for medications, tests, and investigations. Thus, if you can't afford, you'll feel miserable, your symptoms will get worse, and you might even die.

<Files\\Data\\P13> - § 1 reference coded [0.91% Coverage]

Reference 1 - 0.91% Coverage

I am dependent on my family because I can't afford to buy.

<Files\\Data\\P14> - § 1 reference coded [1.18% Coverage]

Reference 1 - 1.18% Coverage

I am waiting financial support from my brothers. This is really disturbing.
