# Supplementary material for: The experience of people living with heart failure in Ethiopia: A qualitative descriptive study
Source: PLoS One. 2024 Oct 24;19(10):e0310600. doi: 10.1371/journal.pone.0310600 (PMC11500853; doi:10.1371/journal.pone.0310600)
Supplement: Attachment — Submitted filename: Comments to Authors.docx [file pone.0310600.s002.docx]

Comments to Authors

I believe the manuscript is an important study that contributes to the existing evidence on the experience of people living with heart failure and its impact on quality of life. However, some points need to be elaborated further by the authors to ensure the paper's clarity.

\# I would suggest the title need to be modified ‘Experience of people living with heart failure in Ethiopia: a qualitative study’

#Abstract

*Background

I would suggest using full wordage for heart failure in line 33, page 2, as abbreviations are not recommended in an abstract, as all abbreviations should be defined in the background section. Therefore, it is best to remove all abbreviations in brackets in the abstract and only use full wordage.

Line 37, page 2 you can write as ‘A descriptive qualitative ….’

* Result

I would suggest that to increase the transparency and attractiveness of the manuscript. In the results report, the number and list of categories, themes, and sub-themes should be indicated in both the abstract and the text. And the authors should also enclose the themes and subthemes in the quotation marks across the manuscript.

* Line 53, page 2, “People living with HF in Ethiopia experience various symptoms.” What are various symptoms? Based on the authors manuscript findings, the above sentence doesn’t quite inline.

* Line 53-55, page ‘The impact of HF on various aspects of their lives, combined with poor general health perceptions, significantly affects their HRQoL.’ I could not find any mentions ‘….poor general health perceptions in your findings. Could you clarify whether it is a health care providers or patients who have these perceptions.

* Line 55, page 2, ‘They’ represents ……

*Line 56 and 57, page 2, I could not find any mentions ‘……to understand their experiences and impact on their daily life’ in your findings.

* …what are ‘Effective multimodal interventions…..’

I suggest the authors modifying conclusion based on their pertinent findings.

#Keyword, use Mesh terms

#Background

*In the first sentence you explained the prevalence in developed, whereas in the same sentence you discussed quality data in developing countries. It needs to be rewritten.

* Line 66, page 3, ……reason for hospitalisation. It needs to be cited.

* Line 67, page 3, …… within family and community. It needs to be cited.

* Line 71, page 3, …… unpredictable nature of the disease. It needs to be cited. Line 74, page 3, …… and palpitations. It needs to be cited.

* Line 75, page 3, …… as depression and anxiety. It needs to be cited.

*Line 76, page 3, I would suggest using full wordage for HRQoL, the first time it appears in the background section.

*The fourth paragraph needs to be merged with the second paragraph, as both paragraphs explain HF symptoms and HRQoL.

*Added a little bit HF and HRQoL low resource settings and Ethiopia.

*Line 88, page 3…….in Ethiopia (18, 19). Both references do not explain the HF is a significant problem in Ethiopia. what study gaps do authors want to uncover?

#Methods

* Sampling and recruitment

*Who distributed invitation letters to all eligible participants?

*How to compensate participants for their time when the authors invite them to participate the study?

*Participants were recruited using a purposive sampling technique. according to what criteria?

*How did you employ this purposive sampling technique? with who? By whom?

*…..interview guide developed using the TSM. Who was the team that developed it?

*Signs of data saturation were observed after 11 interviews. You used descriptive qualitative approach and used ‘data saturation’ rather than ‘information power’ for sample size determination. ‘Data saturation’ is recommended for grounded theory, but ‘information power’ is recommended for descriptive qualitative approach. Therefore, you should a little bit justify it.

Q#4 is a leading question

*The interviews were imported into NVivo version 12 ‘statistical software’ for analysis?

*creating new themes inductively, reviewing themes, defining, and naming themes, and producing the final report (31). Use the updated reference and phase.

\# Results

*Were participants given a chance to review their transcripts/ summary of findings?

*Line 207, page 8, it is not clear to me ‘Experiences of people living with heart failure’.

*Authors need to write themes and subthemes as word enclose quotation mark rather than number.

*Themes are broadening idea some are overlapped It can be merged.

*Perception of HRQoL and its influencing factors … needs revision.

*It is important for ‘Initial reaction to diagnosis’ subthemes is resynthesis and see quotes.

*Line 232, page 10, …. Most of the participants. There is one quotation, how do say most of the participants?

*The same issue …. line 248, page 10… Many participants……..

*Similar ideas and quotes appear in multiple places. For example participants reported feeling shocked, anxious in the ‘Emotional symptoms’, ‘Initial reaction to diagnosis’ , ‘Emotional impact’ and ‘Factors influencing HRQoL’ subthemes.

*Line 446, page 17, ‘Lack of continuity of care’. It needs revision

*Routine mental health evaluations and counselling as part of HF management could be beneficial. How do you come up in this conclusion?
